# Mode II Delamination under Static and Fatigue Loading of Adhesive Joints in Composite Materials Exposed to Saline Environment

**DOI:** 10.3390/ma16247606

**Published:** 2023-12-12

**Authors:** Paula Vigón, Antonio Argüelles, Miguel Lozano, Jaime Viña

**Affiliations:** 1 Department of Construction and Manufacturing Engineering, University of Oviedo, 33203 Gijón, Spain; vigonpaula@uniovi.es (P.V.); antonio@uniovi.es (A.A.); lozanomiguel@uniovi.es (M.L.); 2 Department of Materials Science and Metallurgical Engineering, University of Oviedo, 33203 Gijón, Spain

**Keywords:** composites materials, adhesives, fatigue, fracture, mode II

## Abstract

This study investigates the fatigue delamination behavior of adhesive joints in epoxy carbon composite materials under Mode II fracture loading. The joints were characterized using the End-Notched Flexure (ENF) test, comprising adhesive joints formed by bonding two unidirectional carbon fiber epoxy matrix laminates with epoxy adhesive. These joints were subjected to different exposure periods (1, 2, 4, and 12 weeks) in a saline environment. Prior to dynamic fatigue testing, critical Mode II energy release rate values were determined through quasi-static tests, serving as a reference for subsequent fatigue characterization. This study aimed to comprehend how exposure duration to a saline environment affected the initial stage of fatigue delamination growth and employed a probabilistic model based on the Weibull distribution to analyze the experimental data. The results, gathered over a two-year experimental program, revealed varying behaviors in adhesive joint resistance to delamination based on exposure duration. A noteworthy reduction in fatigue strength capacity was observed, with fracture energies for infinite fatigue life reaching approximately 20% of their static loading capacity. This study sheds light on the deterioration of adhesive joints when exposed to a saline environment.

## 1. Introduction

Fiber-reinforced composite materials are gaining prominence across various industrial sectors due to their advantageous properties compared to conventional metal-based materials. The equivalent mechanical properties and ease of molding of these lighter materials offer substantial weight savings and, consequently, fuel efficiency gains, contributing to the sustainability of various industrial products. Carbon fiber-reinforced polymers (CFRPs) enable the design and manufacture of lighter components, reducing fuel consumption and greenhouse gas emissions without compromising strength and rigidity. These properties have led to their increased use in various applications including automotive, marine, military, aerospace, and renewable energy (such as wind turbine blades).

However, despite their advantages such as high mechanical strength [1,2] and corrosion resistance, CFRPs still exhibit low resistance to delamination, which remains a limiting failure mode in these materials. Therefore, alternative bonding techniques, such as adhesive bonding, are being researched to address this limitation. Adhesive bonding offers several advantages over mechanical fastening methods: more even load distribution over a larger area, elimination of stress concentration points caused by drilling holes, absence of visible fasteners, improved sealing, significant reduction in joint weight, and the ability to bond composites with other materials like metals.

In adhesive bonding, when studying the adhesion phenomenon in relation to delamination in composite materials, it is essential to take into account both the physical, chemical, and mechanical properties of the bonded materials. Additionally, surface preparation of the composite substrates is crucial [3,4] as it makes the adhesive surface receptive to forming strong and durable bonds [5], especially in fiber-reinforced polymers due to their low surface tension and wettability. It is worth noting that these types of bonds result in an increase in the energy dissipation capacity during adhesive joint fracture [6]. Quality assessment of the bond is determined through mechanical tests that induce crack growth in the adhesive joining the two substrates [7,8].

Most studies on adhesive joints in composite materials have primarily focused on epoxy adhesives, although there are now studies on acrylic adhesives [9,10], which exhibit a more ductile behavior compared to the generally stiffer and more brittle epoxy adhesives.

Another important parameter is the influence of the type and rate of applied load [11]. Moreover, research is ongoing regarding the behavior of adhesive joints concerning initiation and growth of delamination. These studies examine joint properties, adhesive thickness [6], and the use of various types of adhesives [12,13].

Other research areas in this field involve analyzing the behavior of adhesive joints under different fiber orientations in composite materials [14] and their response to various degradation processes, including humidity, exposure to a saline environment [15,16], freezing and thawing cycles [17], temperature effects [18,19], and the combined effects of temperature and humidity [20].

It is essential to highlight the relationship between material behavior in an inert environment and the same material subjected to different aging periods, influenced by temperature, humidity, and pressure [21,22,23,24,25]. These factors can modify the static and fatigue strength of composites and are crucial to consider when designing elements using these materials.

Additionally, significant experimental research is being conducted to modify the properties of the adhesive bond by incorporating additional elements aimed at enhancing adhesion or acting as reinforcements against delamination. The addition of carbon nanotubes (CNT) [26,27] or functionalized-graphene nanoplatelets (f-GNPs) allows us to see an increase in electrical impedance when the conductive network in epoxy matrix breaks at the front of the crack [28].

Studying fracture behavior in its different pure modes under fatigue loading on fiber-reinforced composite materials is a complex phenomenon that requires thorough investigation. While research into Mode I fracture [29,30] has received significant attention from various authors, Mode II fracture, whether under static or dynamic loading, has been less studied, and there is limited literature available [31,32,33,34]. The fracture in this mode occurs when surfaces slide laterally against each other in parallel directions; this is called shear fracture. This mode of fracture is important in composite materials elements. For this mode, the most commonly used testing methodology is the End-Notched Flexure (ENF) test, which assesses interlaminar fracture toughness in laminated composites or adhesive joints and has been standardized by ASTM D7905/D7905M–14 [35] for Mode II (G_IIC_). Within the realm of composite materials, delamination becomes a paramount design concern. Assessing interlaminar fracture resistance, particularly in Mode II, is a critical factor in product development and material selection within this context. Having a measure of interlaminar fracture toughness that is independent of sample geometry and force application method is highly advantageous, as it enables the establishment of permissible values in damage tolerance analysis for such materials. Considering both the uncracked and cracked toughness facilitates appropriate selection based on specific application requirements. The method proposed in [35] serves various purposes, including quantifying the effects of fiber surface treatment and local variations in fiber volume fraction, as well as processing variables and environmental conditions on the G_IIc_ value of a particular composite material. Furthermore, it is valuable for the quantitative evaluation of G_IIc_ values obtained from different batches of a specific composite material, which can be highly relevant for material selection. Ultimately, this test method contributes to the development of delamination failure criteria and the assessment of composite structure durability.

In addition to this study of Mode I and Mode II, situations of mixed mode are also investigated, involving a combination of stresses from both modes [36,37,38,39]. This analysis offers a more comprehensive understanding of the behavior of adhesive joints under a wide range of loading conditions.

Beyond relying solely on experimental methods, the utilization of finite element analysis and the structural assessment of components subjected to various loading conditions, especially when they are composed of composite materials bonded with adhesives, becomes imperative. Researchers have been dedicated to acquiring the requisite knowledge for numerical simulations, paving the way for the development of novel methodologies and the pursuit of computational solutions for this unique class of joints [40,41,42].

The objective of this study has been to evaluate the behavior of adhesive joints concerning the fatigue delamination phenomenon under Mode II loading when subjected to different exposure periods in a saline environment. For this purpose, a unidirectional carbon fiber-reinforced epoxy matrix composite material was selected as the substrate, along with an epoxy-based adhesive. The rate of energy relaxation achieved by the joint under Mode II fracture loading was taken as this study’s parameter for characterizing delamination resistance. This study analyzes how the degradation process, in relation to the exposure periods it has undergone, influences adhesive joints and their fatigue life during the initiation phase. To better interpret the experimental results obtained, they have been processed using a probabilistic model based on a Weibull distribution [43].

There is a scarcity of valuable data for the design of resilient components that offer insights into fatigue behavior when the material is subjected to this kind of degradation. This work contributes valuable information for industrial components exposed to dynamic loads operating under similar conditions, such as offshore wind turbines.

## 2. Materials and Methods

### 2.1. Materials Used

The materials used are described below, including the type and basic characteristics of the composite material employed, as well as the type of adhesive used in this study.

#### 2.1.1. Type of Composite Material Used

The substrate used in this study is a composite material featuring an epoxy matrix and unidirectional carbon fiber reinforcement, known commercially as MTC510-UD300-HS-33% RW. Table 1 provides an overview of the laminate’s mechanical properties. The manufacturing process, which serves as the foundation for this research, entailed molding and vacuum consolidation, following the recommended thermal curing cycle specified by the prepreg manufacturer. The fibers in the composite were aligned in a unidirectional configuration at 0°.

#### 2.1.2. Adhesive Characteristics

The adhesive joints in this study utilized a commercially available epoxy-based adhesive named Loctite^®^ EA 9461. Table 2 presents the properties of this adhesive. The bonding material’s surface was pre-treated. Upon completion of the adhesive curing cycle, the laminate was machined to obtain the test specimens, with final dimensions as follows: width of 20 mm and length of 225 mm, with a crack initiation length of 60 mm from the the end of the specimen. The total thickness of each specimen was 4.3 ± 0.1 mm. A 12 μm thick PTFE (Polytetrafluoroethylene) anti-adhesive film was placed between the substrates at one of their ends and served as an initiator for the delamination process.

## 3. Experimental Methodology

This section describes the key elements of the experimental program carried out to assess the adhesive bonding of the selected composite material in relation to delamination under Mode II fracture conditions. It covers both static and fatigue conditions under different exposure durations in a saline environment.

### 3.1. Surface Conditioning

The composite material used as the substrate was prepared by manually sanding the surface with Al_2_O_3_ sandpaper with a grain size of P220. After treating the composite surface, it was cleaned and degreased in preparation for the subsequent bonding process.

### 3.2. Environmental Degradation Processes

The purpose of the degradation processes, known as aging, used in this study was to evaluate the quality of the adhesive bond over time and the effects of different external agents (humidity, temperature, and saline concentration). To do this, various parameters were selected in a salt spray chamber to accelerate the impact of these external agents.

The external agents considered can affect both the adhesive-substrate interface and the individual components. Understanding the impact of these external factors on the bond allows for predicting its behavior in service, assists in the proper selection of the materials that compose it, and helps find solutions to potential problems that could increase its industrial implementation costs.

For the accelerated simulation of the aging process in a saline environment, a Köheler brand salt spray chamber, model DCTC 1200 P, was used. The environmental conditions considered included an average indoor temperature of 35 °C ± 2 °C, relative humidity of 89%, pressure of 1.2 bar, and a saline solution prepared by dissolving “p.a” quality sodium chloride (reactive for analysis) in demineralized distilled water with a concentration of 50 g/L. This solution had a relative density between 1.0255 and 1.04 g/cm^3^, pH between 6.5 and 7.2, and a flow rate between 1 and 2 mL/h. Throughout the entire process in the chamber, the parameters remained constant. At the end of the process, the samples were removed and any residues from the saline solution were discarded. The exposure times selected for the salt spray chamber were 1, 2, 4, and 12 weeks.

### 3.3. Characterization of Material Behavior against Delamination

#### 3.3.1. Static Characterization

**Figure 1:**
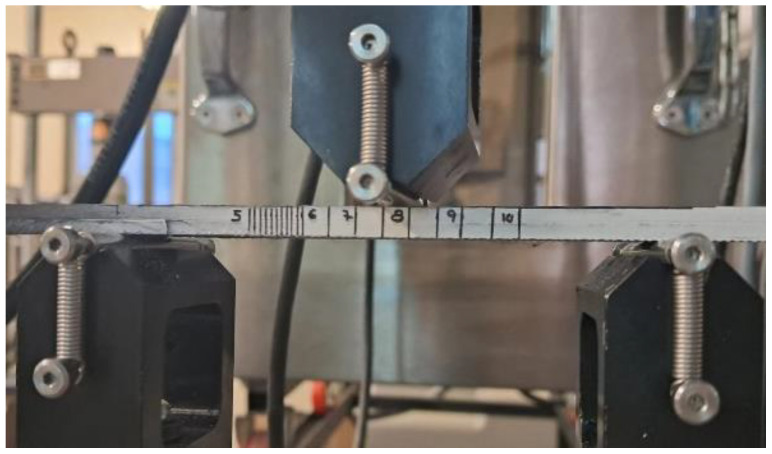
Specimen Placement on Testing Equipment.

To examine the impact of varying aging durations on the delamination phenomenon in static conditions, the Mode II fracture energy release rate was investigated. Figure 1 shows the tests that were performed on ENF-type specimens, following the testing procedure outlined in ASTM 7905/D7905M–14 [35]. To determine the Mode II fracture energy release rate (G_IIC_), experimental calibration of the flexibility (NPC) was employed, as defined by the formula presented in Equation (1).

G_IIC_ = 3ma_0_^2^P_máx_^2^/(2B) (1)

where ‘B’ symbolizes the width of the specimen, while ‘P_max_’ represents the maximum applied load. The term ‘a_0′_ refers to the initial crack length, and ‘m’ is a function of the specimen’s flexibility. This flexibility is experimentally obtained by loading and unloading the specimen within the elastic zone. During this process, load and displacement values are recorded at various positions of the crack front relative to the nearest support. The value of ‘m’, which signifies the specimen’s flexibility, is determined based on the slopes obtained.

Following the established methodology, static characterization was carried out for all the exposure periods under study. The findings are encapsulated in Table 2, which can be found in the section dedicated to experimental results.

#### 3.3.2. Fatigue Characterization

The experimental fatigue program was designed with the objective of discerning the fatigue behavior of the adhesive bond when it was subjected to a delamination process under Mode II fracture and dynamic loading. The aim was to quantify the potential influence of varying exposure periods in the salt spray chamber on its response to this phenomenon. In the analysis of the fatigue delamination initiation phase, this study defined fatigue failure as the point at which interlaminar crack propagation commenced. The fatigue limit for testing was established at two million cycles.

These tests were carried out in accordance with the guidelines stipulated in ASTM D3039M-17 [34], a standard originally developed for Mode I fracture but adapted in this instance for Mode II. Five constant loading levels were implemented, which were determined based on the values derived from the prior static material characterization for each exposure period they underwent. This was supplemented with isolated tests to enrich the experimental data. The definition of these loading levels took into account the results obtained from the previous material characterization under static conditions, with these levels calculated as percentages of the critical Mode II fracture energy release rate, G_IIc_. All fatigue tests were conducted with an asymmetry coefficient of R = G_min_/G_max_ = 0.1 and displacement control on the testing equipment.

## 4. Results and Discussion

The experimental results derived from the analysis of the behavior of adhesive bonds under Mode II fatigue loading, which were subjected to various aging periods, are presented herein.

### 4.1. Static Regime

Table 3 showcases the mean values of the Mode II fracture energy release rate (G_IIC_), maximum load (P), and displacement (δ) at the maximum load point for the various aging periods considered. From the results obtained, it can be discerned that there is a marginal decrease in the average displacement achieved by the tested samples as the exposure period in the chamber lengthens. This implies a minor reduction in the ductility of the adhesive as the exposure period extends. However, when considering the maximum load level reached, nearly constant values are obtained irrespective of the duration of exposure.

Using the Mode II fracture energy release rate G_IIC_ as a reference parameter and comparing the values achieved by the adhesive bond exposed to different periods of time in a saline atmosphere with the material without exposure, a significant increase in this parameter is observed for two and four weeks of exposure. Values close to the initial values are obtained after a 12-week exposure period, which is approximately 8% lower. These values have been used as a reference for the subsequent definition of the fatigue testing strategy.

### 4.2. Dynamic Regime

To enhance the reliability of result assessment, a probabilistic analysis of the entire fatigue life range was performed, for which various models are available. In this study, a Weibull regression model proposed by Castillo et al. [43] was employed as a statistical tool. This model facilitates the normalization of the entire fatigue life range and has previously demonstrated its efficacy in other composite materials applications.

The choice of this model is justified by the research team’s prior experience in applying it to fatigue behavior in various types of composite materials. It has provided valuable insights by enabling the normalization of the entire fatigue life range under study, thus reducing errors and minimizing the impact of variability associated with the behavior of these composite materials, particularly under fatigue conditions. Additionally, it allows the determination of the material’s fatigue life at values beyond what can be achieved through testing. This information is crucial for industrial design, as it enables the estimation of fatigue life beyond 10 million cycles, resulting in significant time savings during experimental characterization.

**Figure 2:**
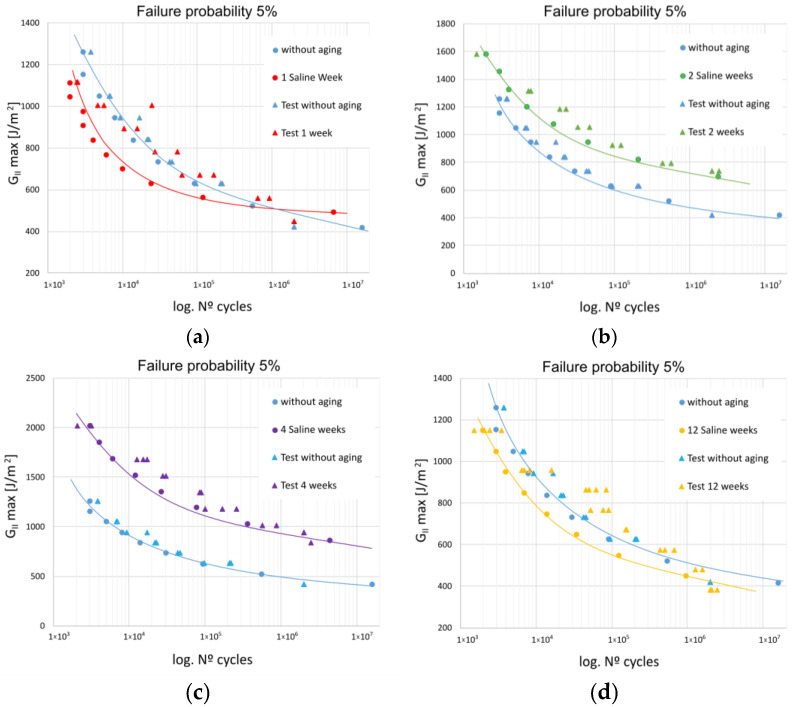
Comparative fatigue initiation curves between experimental results without exposure and different aging periods and Weibull fitting: (**a**) Unexposed specimens and 1 week of saline exposure; (**b**) Unexposed specimens and 2 weeks of saline exposure; (**c**) Unexposed specimens and 4 weeks of saline exposure; (**d**) Unexposed specimens and 12 weeks of saline exposure.

Figure 2 shows the fatigue delamination initiation curves under Mode II fracture. It presents the maximum Mode II fracture energy release rate (G_IIC max_) as a function of the number of fatigue life cycles. Each of the graphs within the figure compares the different exposure periods of the material in a salt spray chamber: one week (Figure 2a), two weeks (Figure 2b), four weeks (Figure 2c), and twelve weeks (Figure 2d), with the results obtained without exposure to a saline environment, at a 5% fatigue failure probability. Additionally, the experimental results are also presented.

The obtained results indicate that when using the fatigue limit as a reference parameter and considering the energy release rate at which this limit value is reached for the different analyzed exposure periods, some variability is observed among them. This suggests that the exposure periods may significantly impact the fatigue limit and energy release rate due to the breakage of some of the bonds in the polymer chain.

For the case of the unexposed material (no aging), the energy release rate for infinite fatigue life is approximately 405 J/m^2^. This is very close to the values obtained for exposure periods of 1 week (500 J/m^2^) and 12 weeks (390 J/m^2^). In contrast, exposure for 2 weeks results in energy release rates for infinite fatigue life of 630 J/m^2^, while for 4 weeks, it increases to 810 J/m^2^. These values are nearly double the values obtained for the unexposed material. This suggests that the applied degradation process has a favorable effect on the studied adhesive bond during short exposure periods, possibly due to post-curing of the adhesive, which enhances its fatigue properties.

When examining the complete fatigue life range of the studied adhesive bond for the selected aging periods, two observations can be made. Firstly, there is a more even distribution of the experimental results for the unaged material. Secondly, the improved fatigue behavior of the material exposed to the considered saline environment is confirmed, particularly for exposure periods of 2 and 4 weeks.

**Figure 3:**
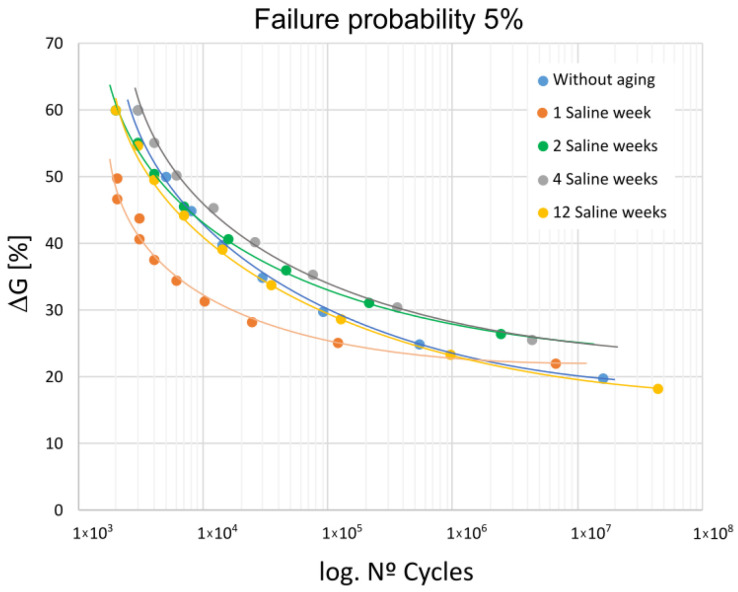
Fatigue behavior for the studied periods, as a function of stress level.

Figure 3 shows the fatigue behavior for all the considered exposure periods, presenting the stress level in %, which has been calculated with reference to the value obtained in the previous static characterization, plotted against the number of cycles.

From the analysis of their behavior, it is derived that the fatigue life limits for the different aging periods considered, as determined by the statistical model used, would be in the range of 20% to 26% of G_IIC_, the energy release rate obtained from the static characterization. These limits are higher for specimens aged for two weeks and four weeks, which practically overlap.

It is worth noting that when considering the entire fatigue life range, not just the fatigue limit, a significant loss of fatigue resistance capacity of the bond occurs for a 1-week exposure period. Fatigue failures occur in the low cycle range, at 50% of its resistance capacity under static loading.

To verify if the adhesive has undergone changes or deterioration in its properties due to the aging process, calorimetry tests [46] were conducted using a Differential Scanning Calorimeter (DSC) equipment SHIMADZU DSC-60. Three samples, with an average weight of 5 mg, were analyzed: one unaged and two aged for 1 and 12 weeks, respectively. The specimens for the calorimetry test were extracted from the adhesive in the specimens tested under Mode II.

**Figure 4:**
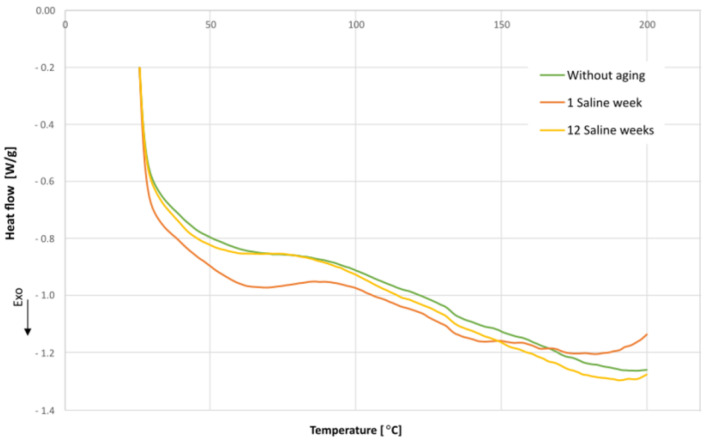
Results of the DSC calorimetry tests.

Figure 4 displays the test results. For the evaluation, the adhesive specimens were exposed to temperatures of up to 200 °C in each period.

The adhesive behavior appears to be altered after one week of aging, correlating with the worst fatigue results, as shown in Figure 3.

**Figure 5:**
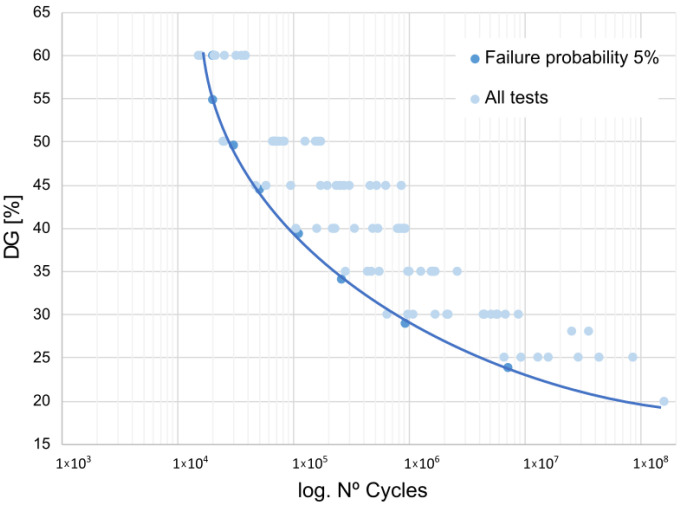
Overall fatigue behavior considering all tests.

Figure 5 shows the comprehensive performance of the examined adhesive bond. It represents all the fatigue tests conducted as a unique and representative sample reflecting the material’s behavior over its entire service life when exposed to a saline environment. The graph displays the stress level ΔG_IIC_ in percentage terms plotted against its fatigue life.

It is observed that the estimated fatigue limit for infinite life, according to the statistical model used for the adhesive bond, would be approximately 20% of the energy release rate obtained from the static characterization. Taking the median of the values derived from the analysis of different exposure periods, this figure amounts to 489 J/m2. It is estimated as the critical energy release rate at which infinite fatigue life would be attained under fatigue loading, irrespective of the environmental conditions linked to saline exposure. This is a relevant data point in the design of industrial components subjected to this type of aging and highlights the severe deterioration that the saline aging process causes in the studied adhesive bond.

### 4.3. Fracture Surface

**Figure 6:**
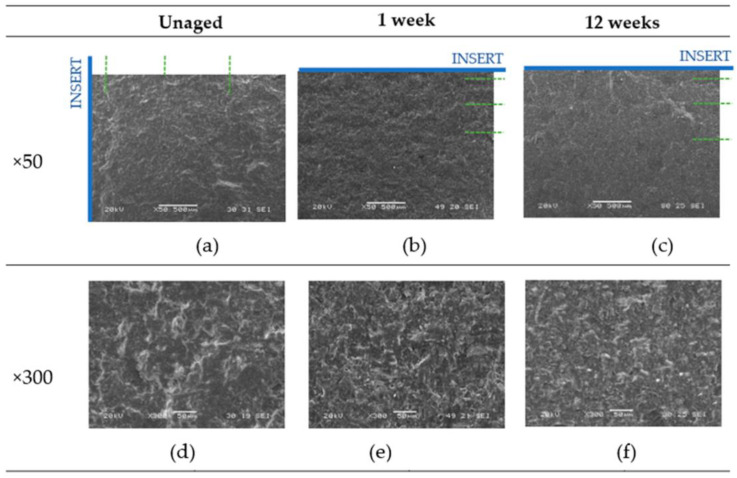
Fracture surface aged and unaged at different magnifications. (**a**–**c**) with ×50 zoom and (**d**–**f**) with ×300 zoom.

**Figure 7:**
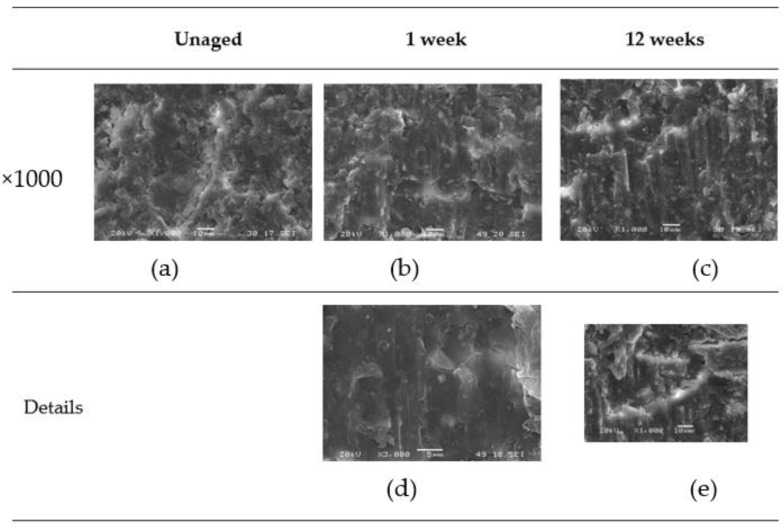
Test surface details. (**a**–**c**) with ×1000 zoom, (**d**) a detail with ×3000 zoom and a detail (**e**) with ×1000 zoom.

Once the samples were tested, the resulting fracture surfaces were analyzed, focusing on the fatigue initiation zone. A scanning electron microscope (SEM) model JEOL-JSM5600 was used. Figure 6 and Figure 7 provide a summary of images considered representative of the surfaces, illustrating the material and adhesive behavior with respect to the type of fracture (mode II) and the aging applied. The analysis was performed at different magnifications: ×50, ×300, and ×1000.

Figure 6 displays images obtained for the unaged material and the material aged for 1 and 12 weeks in the salt spray chamber, with 12 weeks representing the maximum exposure period analyzed. In Figure 6a–c, the direction of fatigue crack propagation and the initiation of delamination (insert) are marked. In all instances, the dominant failure type is cohesive, and the direction of fatigue crack propagation can be observed. On the surface, undulations are generated in the adhesive along its fracture surface (indicated by green dashed lines). It can be seen that the lines generated are more widely spaced in the unaged sample and are more clearly visible, as the adhesive is unaffected by any degradation and provides greater resistance to crack advancement. In contrast, in the other two cases, adhesive degradation affects the clarity of the crack front.

Figure 7 provides close-up details of the samples, offering a more detailed examination of the differences between the unaged and aged specimens.

In general, under mode II fracture, the shear movement between the surfaces can lead to a certain plasticization and sanding of the adhesive in the direction of fracture motion.

## 5. Conclusions

This study investigates several factors influencing the fatigue delamination process in epoxy-based adhesive joints and epoxy matrix laminates reinforced with unidirectional carbon fibers under mode II fracture conditions. The research focuses on their response to prolonged exposure to a saline concentration environment.

- Characterization under static loading in mode II conditions reveals a notable reduction in the maximum displacement as exposure time to the saline environment increases, with longer exposure periods making the adhesive more brittle.
- The rate of energy release under static conditions shows improved performance of the adhesive joints after 4 weeks of exposure to the saline environment. This suggests that thermal conditions within the chamber enhance substrate adhesion through material post-curing. However, after 12 weeks of exposure, the values achieved are lower than those for the unexposed material.
- In the initiation phase of fatigue behavior, various responses are observed for different exposure levels. Longer exposure periods lead to decreased fatigue limits in comparison to unexposed material. In contrast, intermediate exposure periods of 2 and 4 weeks show improved fatigue behavior compared to the 12-week exposure and unexposed material. This trend aligns with the static loading results and is likely attributed to the adhesive’s post-curing during the initial exposure weeks.
- When considering all the tests as a representative sample of the material’s behavior in a saline environment, fatigue limits for infinite life are approximately 20% of its static load-carrying capacity, highlighting the significant deterioration of adhesive joints due to such aging. This emphasizes the importance of protective measures for joints exposed to saline environments.
- In examining the fracture surfaces, notable distinctions are observed between specimens subjected to prior aging in a salt spray chamber and those that were not. The former show signs of plasticization and abrasion in the adhesive, attributed to the aging and deterioration process of the adhesive, although in both cases the surface shows sanding due to the shear movement.
- The data obtained from this study are applicable to various industrial sectors where composite materials have significant applications. For instance, one application is in the field of offshore wind energy generation, where fatigue in a saline environment is a critical factor in design considerations. While this sector is a primary target for the information provided by this study, it is not the only one. The findings can also be relevant in the aerospace industry due to the diverse operating environments of its products, not to mention the naval sector. In all cases, the results highlight the essential need for industrial design to understand how the material behaves under fatigue in the specific environment it will operate in throughout its service life. This necessitates prior experimental studies to confidently determine the useful energy relaxation rate for the estimated component life. Additionally, the adverse effects of the saline environment can be minimized by applying appropriate surface protection such as a gel coating or specialized coatings. Furthermore, it is recommended to conduct regular inspections of these components during their service life.

In summary, this investigation underscores the influence of exposure time to a high saline concentration environment on the fatigue behavior of epoxy-based adhesive joints and substrates reinforced with carbon fibers. The results highlight the complex interplay of factors, including the impact of exposure on both static and fatigue loading, as well as the necessity for protective measures when dealing with adhesive joints in saline environments.

## Figures and Tables

**Table 1:** Properties related to the mechanical behavior of carbon fibers.

	Elastic Modulus ^a^		Tensile Strength ^a^		Shear Modulus ^b^	Shear Stress ^b^
Material	E_11_ (GPa)	E_22_ (GPa)	σ_11_ (MPa)	σ_22_ (MPa)	G_12_ (GPa)	Τ_12_ (MPa)
3MTM DP8810NS	122	8.5	1156	28	5.2	37
CV	8.5%	8%	12.5%	11.8%	9.8%	2%

^a^ ASTM D 3039M [44]; ^b^ ASTM D 3518M [45].

**Table 2:** Adhesive properties.

	Base	Viscosity [mPa·s] (cP)	Elastic Modulus [GPa]	Tensile Strength [MPa]	Shear Strength [MPa]
Loctite^®^ EA 9461TM	Epoxy	150,000 a 250,000	2.758	30.3	13.8

**Table 3:** Mode II Fracture Behavior as a Function of Exposure in a Salt Spray Chamber.

Aging Time	G_IIC_ [J/m^2^]	P_med_ (N)	δ_med_ (mm)
Without aging	2096.55	1945.36	3.7
1 week	2226.03	1821.92	3.67
2 weeks	2630.67	1906.54	3.40
4 weeks	3359.29	2090.10	3.26
12 weeks	1917.64	2022.60	2.90

## Data Availability

Data are contained within the article.
